# Human cytomegalovirus UL138 interaction with USP1 activates STAT1 in infection

**DOI:** 10.1371/journal.ppat.1011185

**Published:** 2023-06-08

**Authors:** Kristen Zarrella, Pierce Longmire, Sebastian Zeltzer, Donna Collins-McMillen, Meaghan Hancock, Jason Buehler, Justin M. Reitsma, Scott S. Terhune, Jay A. Nelson, Felicia Goodrum

**Affiliations:** 1 Department of Immunobiology, University of Arizona, Tucson, Arizona, United States of America; 2 BIO5 Institute, University of Arizona, Tucson, Arizona, United States of America; 3 Vaccine and Gene Therapy Institute, Oregon Health and Science University, Beaverton, Oregon, United States of America; 4 Imanis Life Sciences, Rochester, Minnesota, United States of America; 5 Department of Microbiology and Immunology, Medical College of Wisconsin, Milwaukee, Wisconsin, United States of America; 6 Abbvie, Chicago, Illinois, United States of America; State University of New York Upstate Medical University, UNITED STATES

## Abstract

Innate immune responses are crucial for limiting virus infection. However, viruses often hijack our best defenses for viral objectives. Human Cytomegalovirus (HCMV) is a beta herpesvirus which establishes a life-long latent infection. Defining the virus-host interactions controlling latency and reactivation is vital to the control of viral disease risk posed by virus reactivation. We defined an interaction between UL138, a pro-latency HCMV gene, and the host deubiquitinating complex, UAF1-USP1. UAF1 is a scaffold protein pivotal for the activity of ubiquitin specific peptidases (USP), including USP1. UAF1-USP1 sustains an innate immune response through the phosphorylation and activation of signal transducer and activator of transcription-1 (pSTAT1), as well as regulates the DNA damage response. After the onset of viral DNA synthesis, pSTAT1 levels are elevated in infection and this depends upon UL138 and USP1. pSTAT1 localizes to viral centers of replication, binds to the viral genome, and influences UL138 expression. Inhibition of USP1 results in a failure to establish latency, marked by increased viral genome replication and production of viral progeny. Inhibition of Jak-STAT signaling also results in increased viral genome synthesis in hematopoietic cells, consistent with a role for USP1-mediated regulation of STAT1 signaling in the establishment of latency. These findings demonstrate the importance of the UL138-UAF1-USP1 virus-host interaction in regulating HCMV latency establishment through the control of innate immune signaling. It will be important going forward to distinguish roles of UAF1-USP1 in regulating pSTAT1 relative to its role in the DNA damage response in HCMV infection.

## Author summary

Human cytomegalovirus (HCMV) is one of nine herpesviruses that infect humans. Following a primary infection, HCMV establishes a life-long latent infection that is marked by sporadic, and likely frequent reactivation events. While these reactivation events are asymptomatic in the immune competent host, they pose important disease risks for the immune compromised, including solid organ or stem cell transplant recipients. Defining complex virus-host interactions underlying mechanisms important for viral latency and reactivation is critical to developing novel strategies to prevent CMV disease following reactivation. We show that a viral latency protein, UL138, interacts with the deubiquitinating complex, UAF1-USP1 to enhance and sustain phosphorylation and activation of signal transducer and activator of transcription 1 (STAT1). STAT1 is a major regulator of the antiviral innate immune response and HCMV is well characterized in its ability to evade this response. However, we demonstrate that HCMV commandeers STAT1 through this interaction to restrict viral replication for latency, in part by acting as a viral transcription factor to regulate UL138 expression. The regulation of STAT1 provides a novel mechanism by which HCMV recruits antiviral innate host responses for viral latency.

## Introduction

Viral latency is a remarkable *coup d’etat* that allows for the virus to persist in the face of robust antiviral immunity and is a hallmark of all herpesvirus infections. Latency is defined as a quiescent state of persistence whereby viral genomes are maintained in the absence of progeny virus production. During latency, viral gene expression is quieted, but not completely silenced [1–3]. The β-herpesvirus, human cytomegalovirus (HCMV), establishes latency in hematopoietic progenitor cells (HPCs within the CD34+ subpopulation) and cells of the myeloid lineage [4,5]. HCMV latency is marked by sporadic and frequent asymptomatic reactivation events, which are controlled by the human immune response. In the absence of adequate cellular immunity, such as in the case of solid organ or hematopoietic cell transplantation, HCMV reactivation from latency poses life threatening disease risk [5–7]. HCMV infection, reinfection or reactivation during pregnancy and subsequent transmission to the fetus makes HCMV the primary infectious cause of congenital birth anomalies [8,9]. The benefits or consequences of lifelong persistence of HCMV in otherwise healthy individuals remain poorly defined [10–15]. Understanding the molecular basis of latency and reactivation is important in developing strategies to target or control the latent reservoir of HCMV and reveals fundamental mechanisms at the pinnacle of virus-host co-evolution.

HCMV encodes viral genes important for latency within the unique long *b*’ (UL*b*’) region of the HCMV genome. The UL*b*’ region is a contiguous 15-kilobase region spanning *UL132-UL150*. These genes are largely dispensable for replication in fibroblasts, but strongly impact infection outcomes in hematopoietic and endothelial cells [16,17]. One of these genes, *UL138*, restricts virus replication in CD34+ HPCs and disruption of *UL138* results in a virus that replicates in HPCs in the absence of a stimulus for reactivation. UL138 functions, at least in part, in modulating the trafficking and signaling of cell surface receptors including, epidermal growth factor receptor (EGFR) [18,19], tumor necrosis factor receptor-1 (TNFR1) [20,21] and multidrug resistance-associated protein-1 (MRP1) [22]. UL138 sustains EGFR signaling, which drives the expression of transcription factors that affect viral gene expression from the *UL133-UL138* locus and latency [18], and sensitizes cells to TNF-α for reactivation [23,24]. A gene co-regulated with UL138, UL135, functionally opposes UL138 in the regulation of EGFR trafficking, targeting it for degradation and attenuating signaling for reactivation [25,26], but has not been shown to impact other UL138-regulated receptors. UL138 function suppresses immediate early gene expression and virus replication, giving it pro-latency properties [17,27,28].

In this study, we define an interaction between UL138 and USP1-associated factor 1 (UAF1) and ubiquitin specific peptidase 1 (USP1). UAF1 is also known as WD-repeat domain 48 (WDR48) protein. UAF1 is a scaffold or chaperone protein that is required for the activity of ubiquitin specific peptidases, USP1, USP12 and USP46 [29,30]. UAF1/USP1 regulates DNA repair processes including homologous recombination and translesion synthesis through deubiquitination of proteins FANCD2, FANCI, and PCNA [31–33]. USP1 is also reported to positively regulate innate pathways by enhancing and sustaining phosphorylation of signal transducer and activator of transcription 1 (pSTAT1) [34], an important activator of the antiviral type1 interferon response. We hypothesized that UL138 may commandeer the UAF1-USP1 interaction to regulate pSTAT1 for latency. We find that sustained pSTAT1 during HCMV infection depends on both UL138 and USP1. Additionally, we show that pSTAT1 localizes to sites of viral DNA synthesis and transcription to regulate viral gene expression. Moreover, USP1 and pSTAT1 activity serve as repressors of viral replication for the establishment of latency in CD34+ HPCs. Collectively, our findings provide mechanistic insight into how HCMV has coopted the innate immune response through STAT1 signaling to regulate decisions to enter or exit latency.

## Results

### Defining UL138-UAF1 host interactions

**Fig 1:**
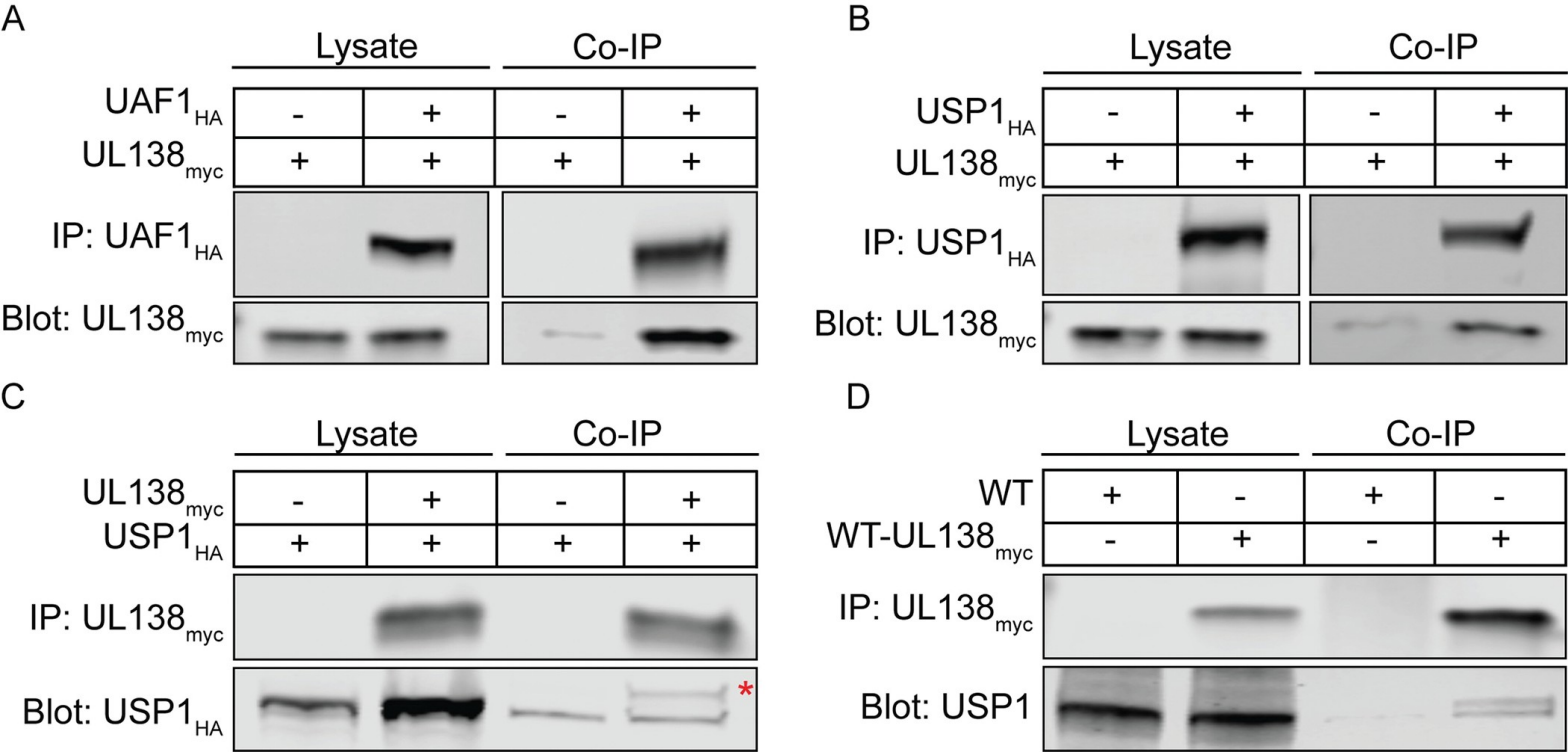
UL138 interacts with UAF1 and USP1. Human Embryonic Kidney (HEK293T) cells were transfected with a UL138_myc_ expressing plasmid along with either an empty vector control plasmid or with (A) UAF1_HA_ or (B) USP1_HA_. At 24 hours post transfection, the HA tag was immunoprecipitated. (C) A reciprocal immunoprecipitation where HEK293T cells were transfected with a USP1_HA_ expressing plasmid along with an empty vector control plasmid or a UL138_myc_ expressing plasmid. UL138_myc_ was immunoprecipitated. The red asterisk marks a higher molecular weight species of USP1 that is enriched with UL138_myc_ immunoprecipitation. (D) Fibroblasts were infected (MOI = 1) with a WT-UL138_myc_ virus. At 48 hpi UL138_myc_ was immunoprecipitated.

We identified host interacting proteins for UL138 by immunoprecipitating UL138 fused in-frame with the Flag epitope tag from fibroblasts infected with the recombinant TB40/E strain, TB40/E-UL138_FLAG_, and defining proteins associated by mass spectrometry (IP-MS/MS). Interacting candidates that precipitated from a control pull down using the FLAG antibody and a lysate from cells infected with TB40/E lacking the FLAG epitope were subtracted from the interacting candidate data set. Top-ranking interacting candidates were based on peptide count and coverage and candidates most pertinent to this work are summarized in Table 1. IP-MS/MS peptides and data are provided for all identified candidates in S1 Table. This screen previously identified the lower-ranking interaction with EGFR [19]. UAF1/WDR48 (herein referred to as UAF1) was the top ranked UL138 interacting protein, followed by WDR20 and USP12. Two previous studies have identified the UL138 interaction with UAF1 (referred to as WRD48), WDR20, and USP12 [35,36], independently validating this work. We validated the UAF1 interaction with UL138 by overexpressing UAF1 fused in frame with a hemagglutinin (HA, UAF1_HA_) epitope tag and UL138 fused in frame with myc epitope tag (UL138_myc_). Immunoprecipitation (IP) of UAF1_HA_ co-precipitated UL138_myc_ (Fig 1A).

USP1 is the most well defined UAF1 interactor, but likely to escape an interactome screen. To determine if USP1 is associated with UL138-UAF1 complexes, we exogenously expressed UL138_myc_ and USP1 fused in frame with a hemagglutinin (HA, USP1_HA_) epitope tag. We detected UL138-USP1 interaction through the co-immunoprecipitation of USP1_HA_ with UL138_myc_ (Fig 1B), as well as the reciprocal IP (Fig 1C). To determine if UL138 and USP1 interact during HCMV infection, cells were infected with a TB40/E-*UL138*_myc_ virus. At 48 hours post infection (hpi), UL138_myc_ IP co-precipitated USP1 (Fig 1D). Immunoprecipitation of UL138_myc_ in both Fig 1C and 1D reveal a higher molecular mass species of USP1 that is enriched in UL138_myc_ pull downs (red asterisk). Therefore, UL138 might interact with a specific post translationally modified USP1 species that is a minor and undetectable species in the lysate and this will be explored further in future studies. While not defined here, the interaction between UL138 and USP1 is likely indirect through UAF1. These results validate the interaction between UL138, UAF1, and USP1.

**Table 1:** Summary of UL138 Interactors^a^.

UL138 Interactor	Cellular Functions	Peptide^b^	Scans^c^	Coverage^d^
WDR48 (UAF1)	Scaffold required for USP1, USP12, and USP46 activity	25	74	39.7
WDR20	Enhances USP12 and USP46 activity; regulates nuclear trafficking of USP12	10	26	20.7
USP12	Sustains pSTAT1 through inhibition of CBP acetyltransferase activity required for dephosphorylation of pSTAT1 by PTPN2	9	26	19.7
EGFR	Activates PI3K/AKT, MEK/ERK, JAK/STAT, and PLC*γ* signaling	8	9	8.9
JAK1	Phosphorylates STAT1	3	3	2.8
UL138	Human Cytomegalovirus early protein that promotes viral latency	8	128	44.9

^a^Candidate interactions determined by IP/MS from infected fibroblasts (TB40/E-UL138_FLAG_) (MOI = 3) at 48 hpi. UL138_FLAG_ was immunoprecipitated with a Flag antibody and following tryptic digest, peptides were identified by IP-MS/MS. Interacting candidates were subtracted from a control Flag pull-down from infected fibroblasts without a Flag tagged UL138. 128 candidates were identified and top interactions were identified by STRING and NCBI analysis. Top-ranking candidates involved in STAT1 signaling are listed based on the peptide count, scan count, and percent coverage.

^b^Number of unique peptides

^c^Number of scans identifying a unique peptide

^d^Percent of the protein covered by the identified peptides

### UL138 sustains pSTAT1 during late times of infection

USP1 has been reported to enhance the activation of STAT1 (phosphorylation on tyrosine 701, pSTAT1) by deubiquitinating and stabilizing tank binding kinase 1 (TBK1) [34]. While the role of UL138 modulating the DDR will be addressed in a separate study, we hypothesized that UL138 may interact with and direct UAF1-USP1 to enhance and sustain pSTAT1 during HCMV infection in order to restrict virus replication.

**Fig 2:**
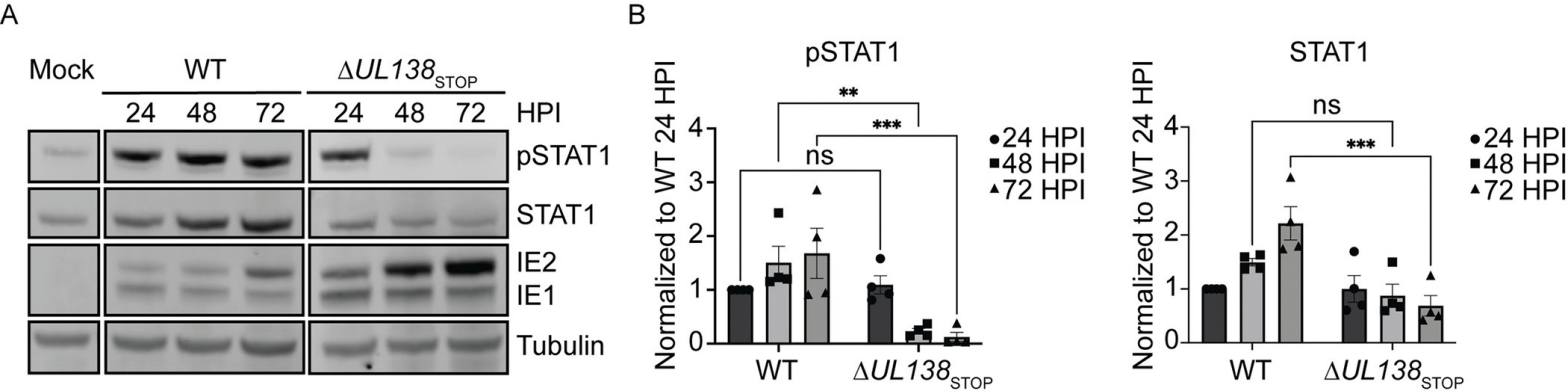
UL138 enhances and sustains pSTAT1 during late times of infection. (A) Fibroblasts were infected (MOI = 1) with a WT or Δ*UL138*_STOP_ virus for 24–72 hpi. At each time point, lysates were lysed and immunoblotted. pSTAT1, STAT1, IE1/2, and Tubulin were detected using antibodies. IE proteins serve as a control for infection and tubulin serves as a control for loading. (B) pSTAT1 and STAT1 were normalized to WT 24 hpi and graphed to calculate statistics. Statistical significance was calculated by Two-Way Anova and represented by asterisks; **p-value <0.01, ***p-value <0.001. Graphs represent the mean of three replicates and error bars represent SEM.

To explore this, we analyzed pSTAT1 in fibroblasts infected with wild type TB40/E (WT) or a virus containing stop codon substitutions in its 5’ end to disrupt UL138 protein synthesis, TB40/E-*UL138*_STOP_ (Δ*UL138*_STOP_) [37]. Over a course of 72 hpi, we observed enhanced and sustained pSTAT1 in the WT infection compared to the Δ*UL138*_STOP_ infection (Fig 2A). pSTAT1 were induced by both infections by 24 hpi, but sustained in WT infection, whereas pSTAT1 was diminished by 48 and 72 hpi in Δ*UL138*_STOP_ infection. This was accompanied by a diminishment in total STAT1 levels in Δ*UL138*_STOP_ infection to resemble uninfected cells, whereas STAT1 remained elevated in WT infection. The induction of total STAT1 in WT infection may be the result of feedback in STAT1 signaling as STAT1 transcripts and protein levels are increased by pSTAT1 signaling [38]. pSTAT1 and STAT1 levels from multiple independent experiments are quantified in Fig 2B. Immediate early (IE) proteins accumulated to greater levels in Δ*UL138*_STOP_ infection relative to the WT infection, consistent with increased IE gene expression and replication of a UL138-mutant virus infection [17,39]. These results indicate that UL138 enhances and sustains pSTAT1 levels during WT infection.

### Late phase induction of pSTAT1 depends on USP1

**Fig 3:**
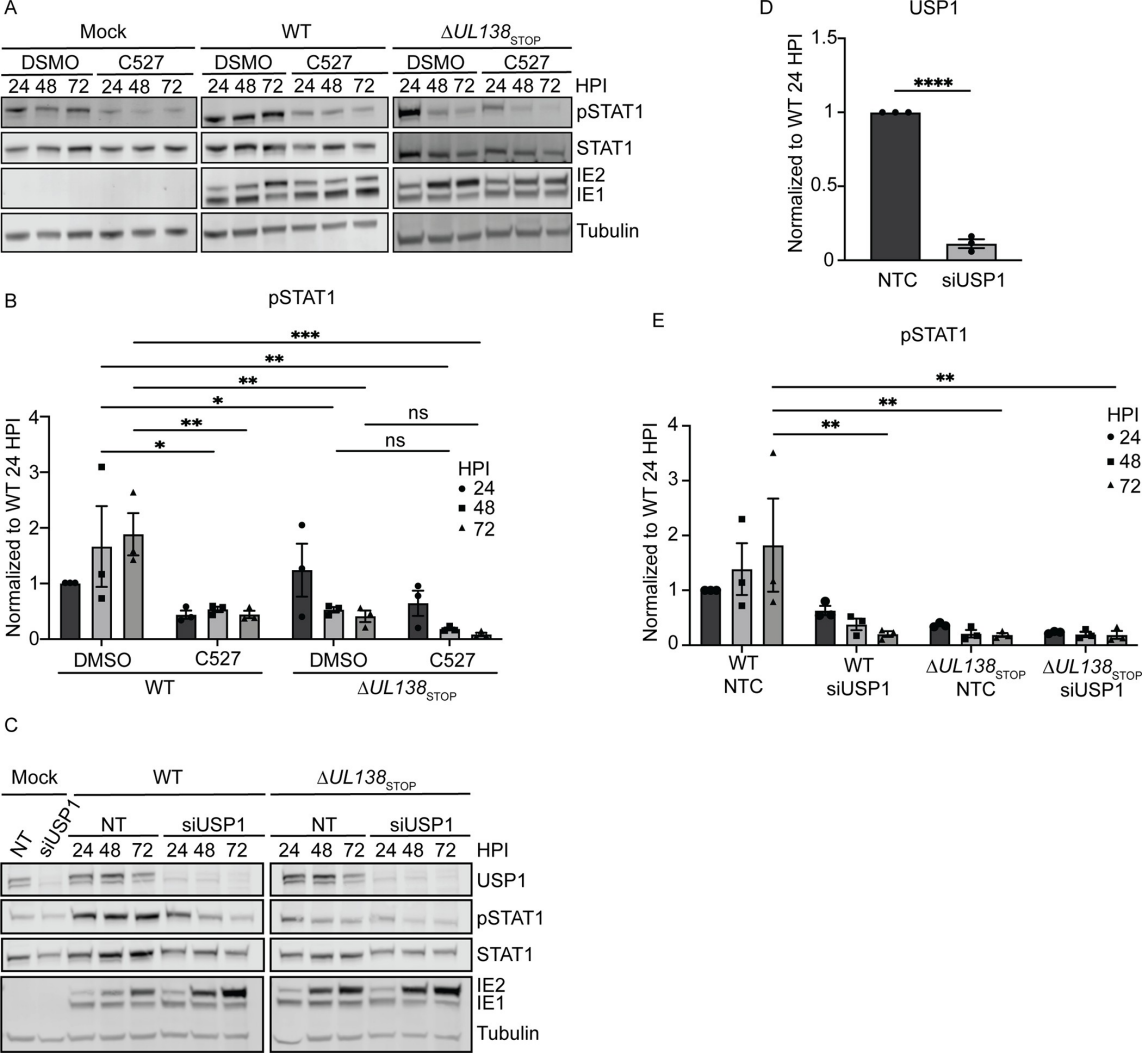
UL138 induction of pSTAT1 depends on USP1 activity. (A) Fibroblasts were treated with either DMSO (vehicle control) or 0.88*μ*M C527 24 hours prior to being infected (MOI = 1) with a WT or Δ*UL138*_STOP_ virus. Lysates were collected and each timepoint and immunoblotted. pSTAT1, STAT1, IE1/2, and Tubulin were detected using antibodies. (B) pSTAT1 was normalized to WT DMSO 24 hpi and graphed for statistical analysis. Statistical significance was calculated by Two-Way Anova and represented by asterisks; *p-value <0.05, **p-value <0.01, ***p-value <0.001. (C) Fibroblasts were reverse transfected with 3 combined siRNA for a non-targeting control (NTC) or USP1. 24 hours post reverse transfection, the media was changed. 48 hours post reverse transfection, fibroblasts were infected (MOI = 1) with either a WT or Δ*UL138*_STOP_ virus and lysates were immunoblotted. USP1, pSTAT1, STAT1, IE1/2, and Tubulin were detected using antibodies. (D). USP1 levels in the NTC and USP1 siRNA knockdown were normalized to NTC and graphed to calculate statistics. Statistical significance for USP1 was calculated using an unpaired t test and represented by asterisks; ****p <0.0001. (E) pSTAT1 was normalized to WT NTC 24 hpi and graphed to calculate statistics. Statistical significance was calculated by Two-Way Anova and represented by asterisks; **p-value <0.01. Graphs represent the mean of three replicates and error bars represent SEM.

To determine if the UAF1-USP1 interaction was involved in UL138-mediated regulation of pSTAT1, we inhibited UAF1-USP1 interactions with the chemical inhibitor, C527 [40] at a low concentration of 0.88 *μ*M which specifically inhibits only UAF1-USP1 deubiquitinating activity [41], in fibroblasts infected with WT or Δ*UL138*_STOP_ viruses (Fig 3A). Relative to DMSO, C527 treatment diminished pSTAT1 levels in cells infected with WT virus but had little effect on Δ*UL138*_STOP_ infection (Fig 3B), suggesting a role for USP1 in the UL138-associated induction of pSTAT1. We further validated the role of USP1 in the UL138-mediated induction of pSTAT1 through siRNA-mediated USP1 knockdown (Fig 3C). Relative to a non-targeting control (NTC) specific to luciferase, we achieved approximately 80% knockdown of USP1 (Fig 3D). Consistent with USP1 inhibition, USP1 knockdown diminished pSTAT1 in WT infection but had little to no effect on pSTAT1 in Δ*UL138*_STOP_ infection (Fig 3E). These results indicate USP1 is also required for sustaining pSTAT1 during HCMV infection.

### UL138-USP1 sustains pSTAT1 independently of TBK1 stabilization

**Fig 4:**
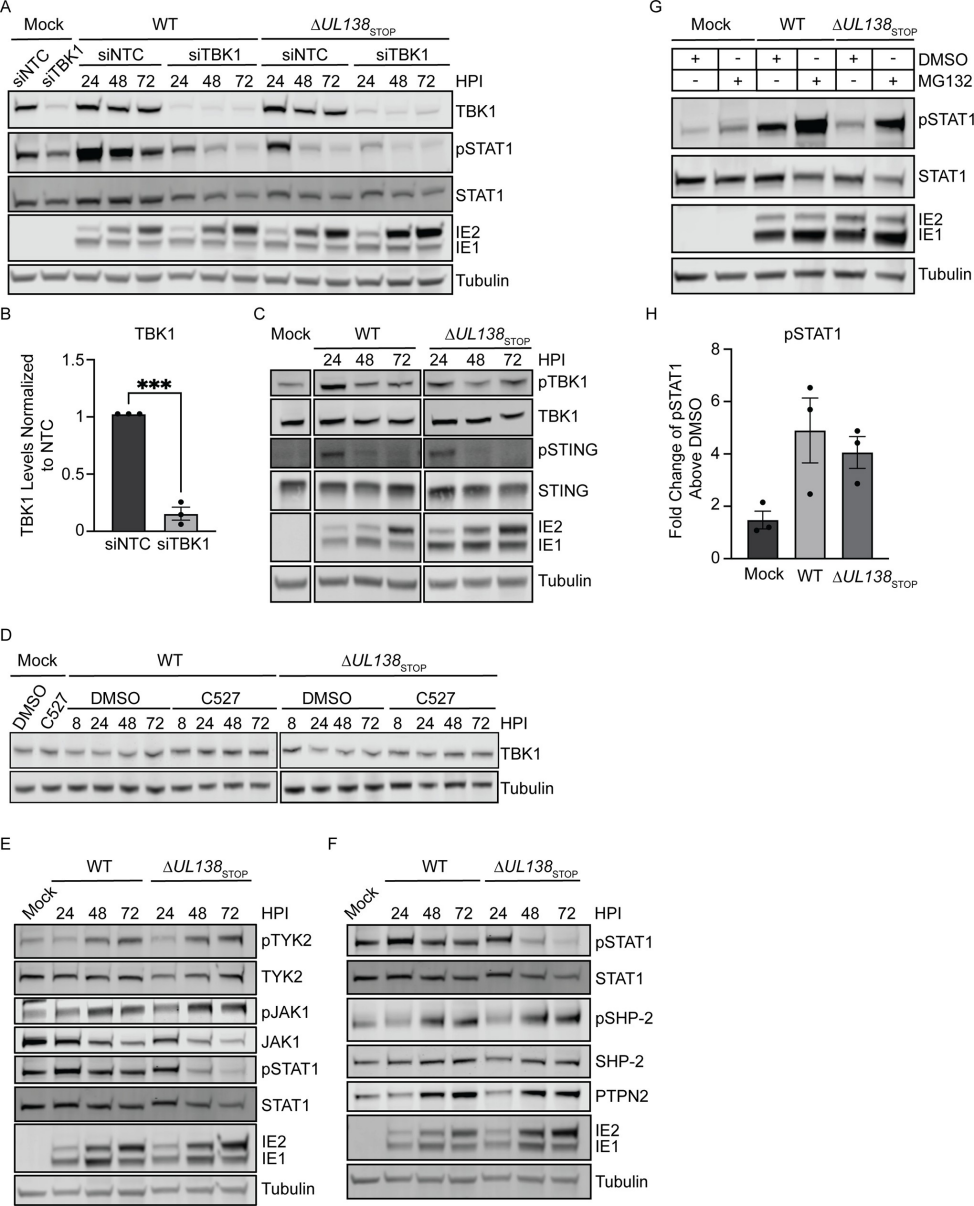
UL138-USP1 regulates pSTAT1 independently of TBK1 stabilization. (A) Fibroblasts were reverse transfected with a siRNA against TBK1 for 48 hours prior to being infected (MOI = 1) with a WT or Δ*UL138*_STOP_ virus. Lysates were collected and immunoblotted for pSTAT1, STAT1, TBK1, IE1/2, and Tubulin. (B) Quantification of TBK1 levels normalized to NTC and graphed for statistical analysis with an unpaired t test and represented by asterisks; ***p-value <0.001. (C) Fibroblasts were infected (MOI = 1) with either WT or Δ*UL138*_STOP_ virus and lysates were immunoblotted. pTBK1, TBK1, pSTING, STING, IE1/2, and Tubulin were detected using antibodies. (D) Fibroblasts were treated with 0.88 *μ*M C527 for 24 hours prior to being infected (MOI = 1) with a WT or Δ*UL138*_STOP_ virus. Lysates were collected and immunoblotted for TBK1 and tubulin. (E) Fibroblasts were infected (MOI = 1) with a WT or Δ*UL138*_STOP_ virus and lysates were collected and immunoblotted for pTYK2, TYK2, pJAK1, JAK1, pSTAT1, STAT1, IE1/2, and Tubulin. (F) Fibroblasts were infected (MOI = 1) with a WT or Δ*UL138*_STOP_ virus and lysates were collected and immunoblotted for pSTAT1, STAT1, pSHP-2, SHP-2, PTPN2, IE1/2, and Tubulin. (G) Fibroblasts were infected with a WT or Δ*UL138*_STOP_ virus (MOI = 1) for 48 hours before treatment with DMSO (vehicle control) or 10 *μ*M of MG132 for 6 hours before lysates were collected. Lysates were immunoblotted for pSTAT1, STAT1, IE1/2, and Tubulin using antibodies. (H) The fold increase in rescue in the MG132 treated cells over the DMSO treated for mock, WT, and Δ*UL138*_STOP_ infected cells were graphed.

To determine if TBK1 is required in the UL138- and USP1- dependent pSTAT1, we knocked down TBK1 with siRNA (Fig 4A). We achieved an 85% knockdown of TBK1 compared to the NTC (Fig 4B). Depletion of TBK1 caused a loss of pSTAT1 in the WT infection and, therefore, the late phase activation of STAT1 is dependent on TBK1 signaling in infection. Given the reported roles of USP1 in stabilizing TBK1 [34] and of UL138 in inducing STING for degradation outside the context of infection [42], we analyzed total and phosphorylated levels of TBK1 and STING during HCMV infection. Phosphorylated and total levels of TBK1 and STING remained unchanged between WT and Δ*UL138*_STOP_ infections (Fig 4C). We further analyzed TBK1 levels during infection with chemical inhibition of USP1 using C527 (Fig 4D) and found no difference in TBK1 levels when USP1 was inhibited. Knockdown of USP1 also failed to alter TBK1 levels (S1 Fig). Therefore, we conclude that UL138-USP1 sustains pSTAT1 independently of TBK1 stabilization.

HCMV infection is reported to induce the proteasomal degradation of janus kinase 1 (JAK1) by unknown mechanisms [43]. We hypothesized that UL138 might block the turnover of JAK1 to sustain pSTAT1 during infection. We analyzed activated and total levels of both kinases upstream of pSTAT1, JAK1 and tyrosine kinase 2 (TYK2) (Fig 4E). Total JAK1 levels decreased throughout HCMV infection but were not differentially regulated in WT and Δ*UL138*_STOP_ infections. Further, total TYK2 levels were unchanged between a WT and Δ*UL138*_STOP_ infection. Levels of phosphorylated pJAK1 (phosphorylated at tyrosine 1022/1023) and pTYK2 (phosphorylated at tyrosine 1054/1055) were induced by 48 hours post infection equally in both infections. Therefore, UL138-UAF1-USP1 maintains pSTAT1 by mechanisms downstream of JAK1 and TYK2 activation.

To terminate STAT1 signaling, pSTAT1 can be dephosphorylated or ubiquitinated for proteasomal degradation in the nucleus. Two phosphatases, src homology region 2-containing protein tyrosine phosphatase 2 (SHP-2) and protein tyrosine phosphatase non-receptor type 2 (PTPN2), are responsible for the dephosphorylation of pSTAT1 [44,45]. We investigated the possibility that UL138 diminishes these phosphatases to sustain pSTAT1. However, during HCMV infection with a WT and Δ*UL138*_STOP_ virus, total levels of PTPN2 and activated levels of SHP-2 (phosphorylated at tyrosine 542, pSHP-2) were induced equally between infections at 48 hpi (Fig 4F). Therefore, UL138 does not alter levels or activation of these phosphatases to regulate pSTAT1.

pSTAT1 has been reported to be targeted to the nuclear proteasome as an additional mechanism to terminate pSTAT1 signaling [46]. We hypothesized UL138 prevents proteasomal degradation of pSTAT1 to sustain pSTAT1 during WT infection. We treated infected fibroblasts at 48 hpi with MG132 for 6 hours to inhibit the proteasome and analyzed pSTAT1 levels in WT and Δ*UL138*_STOP_ infection (Fig 4G). In uninfected cells, MG132 treatment increased pSTAT1 by less than 2-fold over DMSO treatment (Fig 4G). However, upon infection, MG132 treatment rescued pSTAT1 equivalently in both a WT and Δ*UL138*_STOP_ infection with no difference between the ratio of pSTAT1 in MG132 treatment to DMSO in infection. This suggests that the induction of pSTAT1 during HCMV infection is targeted to the proteasome, however, UL138 does not protect pSTAT1 turnover.

### UL138 enhances an early ISG response

**Fig 5:**
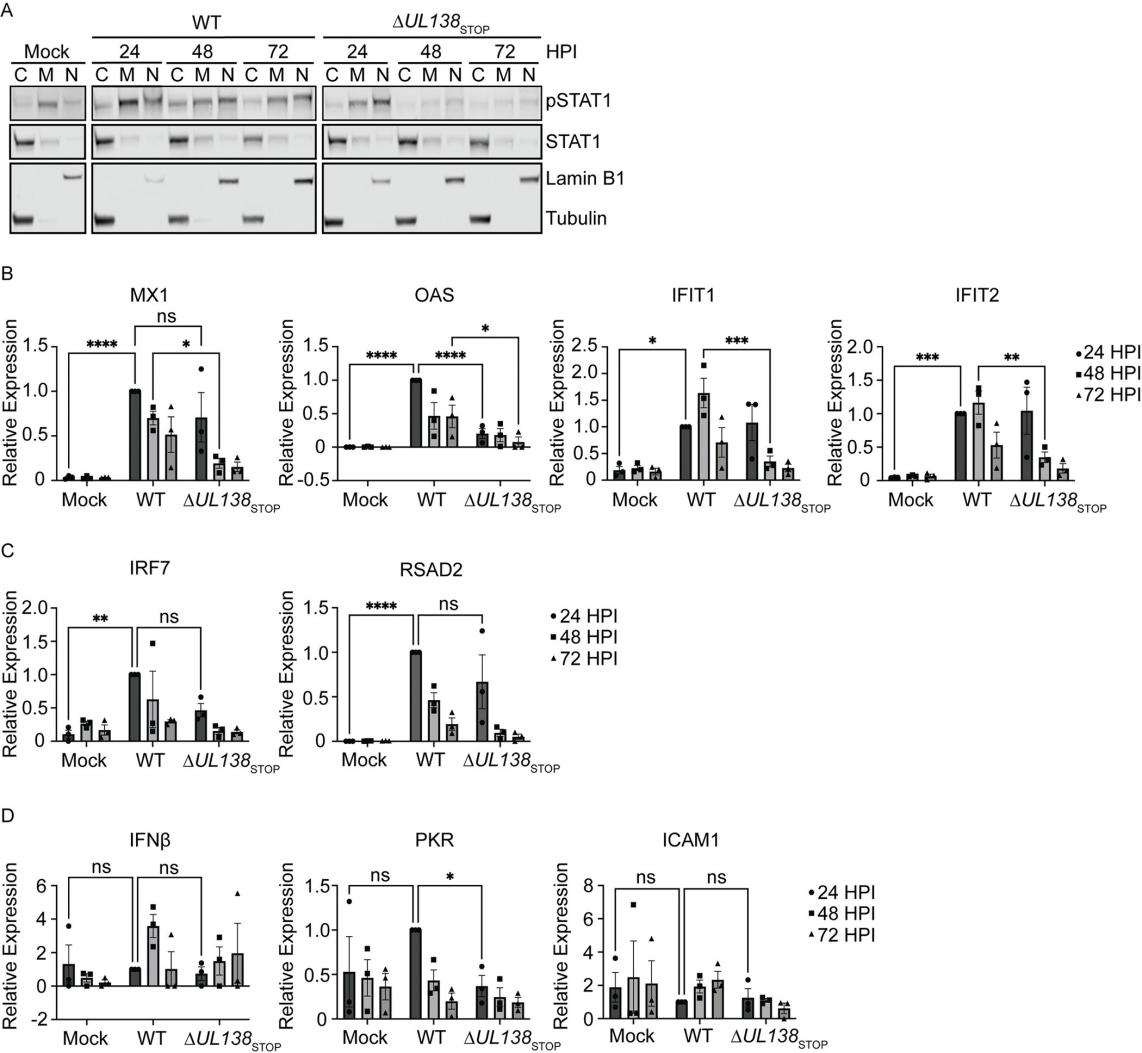
UL138 upregulates an early ISG response. (A) Fibroblasts were infected (MOI = 1) with a WT or Δ*UL138*_STOP_ virus. Cells were fractionated and immunoblotted using antibodies of pSTAT1, STAT1, Lamin B1, and Tubulin. Fraction purity and loading was determined by immunoblotting for Lamin B1 (Nucleus) and Tubulin (Cytoplasm). (B, C, D) Fibroblasts were infected (MOI = 1) with a WT or Δ*UL138*_STOP_ virus. DNA was isolated at 24, 48, and 72 hpi. RT-qPCR for ISG expression was performed with TaqMan Gene Expression Assays (Thermo Fischer Scientific). Relative expression was determined using the ΔCT method and values were normalized to the WT 24 hpi and graphed for statistical analysis. Statistical significance was calculated using Two-Way Anova and represented by asterisks; *p-value <0.05, **p-value <0.01, ***p-value <0.001, ****p-value <0.0001. Graphs represent the mean of three replicates and error bars represent SEM.

Phosphorylation and activation of STAT1 drives its translocation to the nucleus to regulate the expression of hundreds of interferon stimulated genes (ISGs) [38]. Therefore, we next analyzed pSTAT1 localization by subcellular fractionation experiments. pSTAT1 accumulated in the nuclei of cells infected in both WT and Δ*UL138*_STOP_ infection at 24 hpi (Fig 5A). However, pSTAT1 was lost from the nuclear fractions of Δ*UL138*_STOP_-infected cells at 48 and 72 hpi. Therefore, UL138-mediated sustainment of pSTAT1 results in its translocation to the nucleus at late times in infected fibroblasts.

We next analyzed the induction of multiple ISGs directly downstream of pSTAT1 in WT and Δ*UL138*_STOP_ infection by quantitative reverse transcriptase PCR (RT-qPCR). First, we analyzed ISGs that contain an interferon-sensitive response element (ISRE) and therefore, are induced by a type 1 IFN response through the formation of STAT1-STAT2 heterodimers. MX1, OAS, IFIT1, and IFIT2 showed a significantly larger induction in the WT-infection compared to the Δ*UL138*_STOP_-infection either at 24 hpi for OAS or 48 hpi for MX1, IFIT1 and IFIT2 (Fig 5B). IRF7 and RSAD2 were similarly induced in both infections at 24 hpi compared to uninfected cells (Fig 5C). Whereas IFN*β* and PKR were not induced with either infection above uninfected cells (Fig 5D). ICAM1 contains only a gamma activated sequence (GAS) for the binding of STAT1 homodimers when activated by the type 2 IFN pathway [47]. The expression of ICAM1 showed no induction compared to uninfected cells, consistent with a predominantly type 1 interferon response. These results suggest that UL138 induces and sustains specific ISGs in HCMV infection, while several ISGs are induced irrespective of UL138 expression.

### pSTAT1 localizes to sites of viral DNA synthesis and transcription

**Fig 6:**
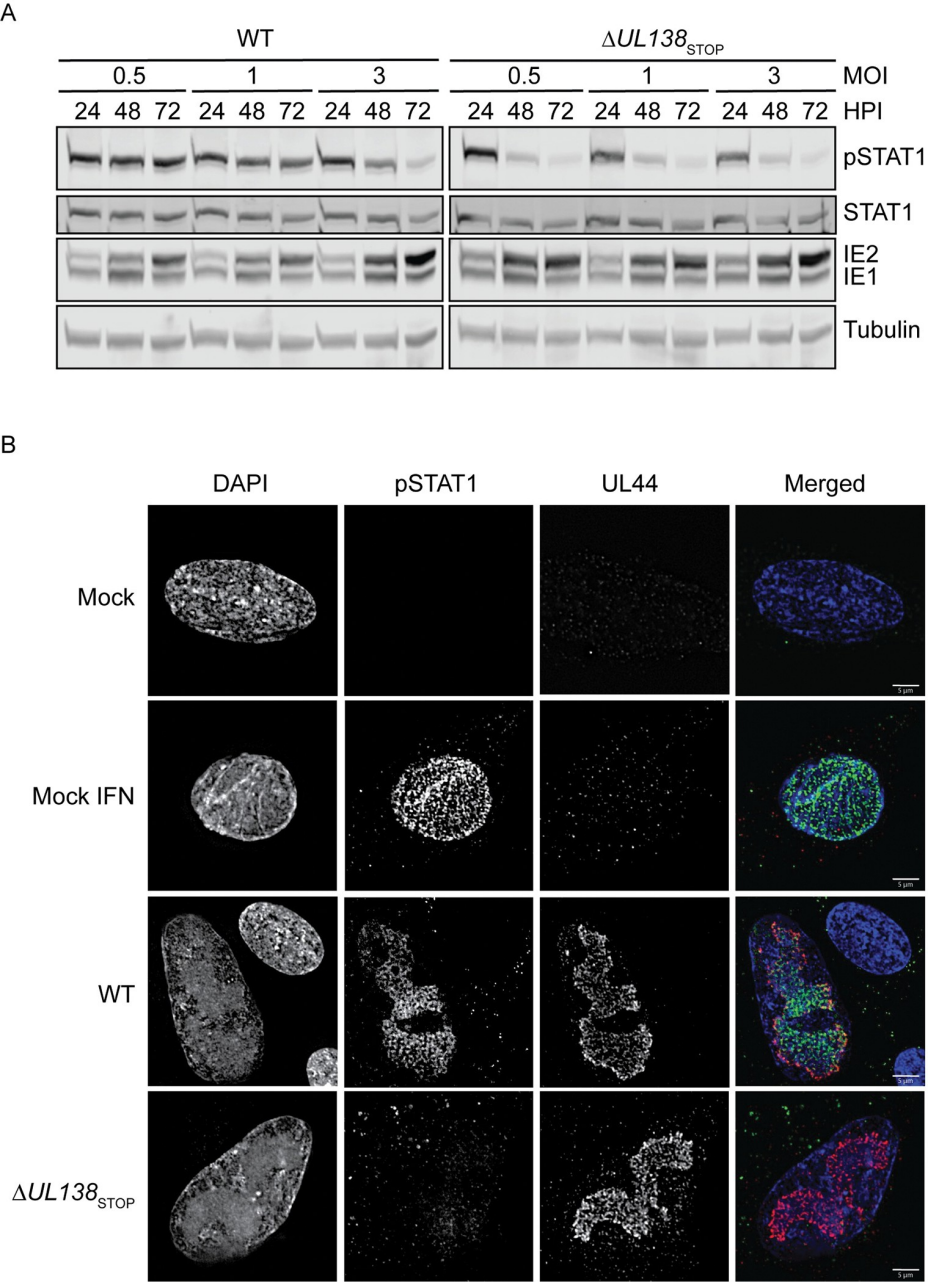
pSTAT1 localizes to sites of viral DNA synthesis and transcription at late times post infection. (A) Fibroblasts were infected at MOI of 0.5, 1, and 3 with a WT or Δ*UL138*_STOP_ virus and lysates were immunoblotted. pSTAT1, STAT1, IE1/2, and Tubulin were detected using antibodies. (B) Fibroblasts were plated on coverslips at 10,000 cells per coverslip. Fibroblasts were infected (MOI = 1) with either a WT or Δ*UL138*_STOP_ virus for 72 hpi. At 72 hpi, mock cells were treated with DPBS or 1000 U/mL of universal type 1 interferon for 30 minutes then the cells were processed per the antibody manufacturer’s recommendations. Coverslips were imaged using a DeltaVision deconvolution microscope.

STAT1 is activated by type 1 interferon paracrine signaling. Therefore, it is possible that the enhanced pSTAT1 detected in WT infection (Fig 2) is indirectly due to UL138 regulation of type 1 interferon secretion and activation of STAT1 in neighboring uninfected cells. To investigate this, we infected fibroblasts with a WT or Δ*UL138*_STOP_ virus at increasing multiplicities of infection (MOI) (Fig 6A). At an MOI of 0.5 or 1, the WT infection augmented pSTAT1 across the 72 hpi time course, although the activation of pSTAT1 was overcome at an MOI of 3 by 72 hpi. Strikingly, the Δ*UL138*_STOP_ infection showed a stark loss of pSTAT1 irrespective of MOI. Collectively, these results suggest that the induction of pSTAT1 signaling does not simply reflect paracrine signaling to uninfected cells. The loss of pSTAT1 in the WT infection at high MOI may reflect the action of multiple viral negative regulators on innate signaling [48] that override UL138-mediated induction of pSTAT1 during a replicative infection, particularly at high multiplicities.

Localization of pSTAT1 to the nucleus during WT infection stimulates production of multiple ISGs early in infection. However, by 72 hpi, many of the induced ISGs return to similar levels seen in uninfected cells independently of the enhanced and sustained pSTAT1 seen at 72 hpi. Therefore, we questioned whether pSTAT1 was localizing to sites of viral DNA replication and transcription at late times of infection. We analyzed the subcellular localization of pSTAT1 in fibroblasts infected with a WT or Δ*UL138*_STOP_ virus at 72 hpi (Fig 6B). Uninfected fibroblasts were untreated or treated with 1000 U/mL of universal type 1 interferon for 30 minutes to induce pSTAT1 localization to the nucleus, as a positive control. In WT infected fibroblasts, pSTAT1 localized within the nuclei of infected cells and co-localized predominantly with UL44, a viral processivity factor that marks replication compartments (RCs), sites of viral DNA replication and transcription. There was no detection of pSTAT1 in a Δ*UL138*_STOP_ infection, consistent with reduced pSTAT1 protein levels (Fig 2). Uninfected neighboring cells in the WT infection showed a lack of induction of pSTAT1 and its localization to the nucleus, consistent with the conclusions that elevated pSTAT1 in a WT infection is not due to paracrine IFN signaling. Colocalization between UL44 and pSTAT1 was detected as early as 48 hpi in WT infection (S2 Fig). From these results, we conclude that UL138 sustains pSTAT1 and that pSTAT1 predominantly localizes to viral RCs. This suggests a role for pSTAT1 in regulating viral gene expression, perhaps to the detriment of cellular ISG expression.

### Late phase pSTAT1 binds to the viral genome and regulates UL138 transcription

**Fig 7:**
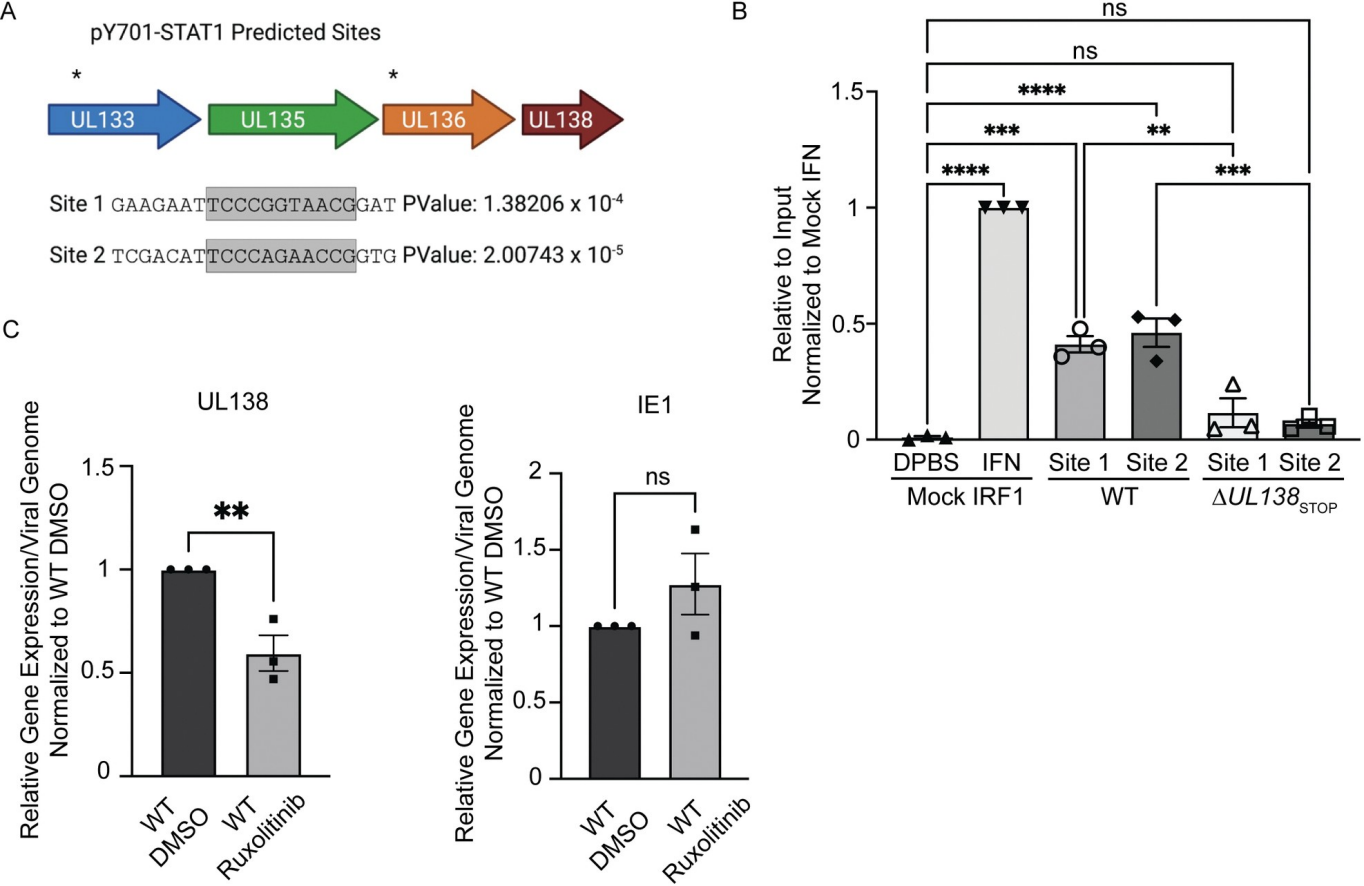
pSTAT1 binds to the viral genome and regulates UL138 transcription during a replicative infection. (A) The HCMV genome was run through PhysBinder Software to obtain potential pSTAT1 consensus sites on the viral genome. Site 1 and 2 are within UL133 and UL136 respectively and are upstream of UL138. (B) Fibroblasts were infected (MOI = 1) with either a WT or Δ*UL138*_STOP_ virus. At 72 hpi, uninfected cells were treated with DPBS (vehicle control) or 1000 U/mL of universal type 1 interferon for 30 minutes. After 30 minutes, all cells entered the CUT&RUN protocol developed by Cell Signaling Technologies. qPCR was performed on the input DNA and immunoprecipitated DNA utilizing primers specific to pSTAT1 binding sites of interest. Percent Immunoprecipitated was calculated to 2% of the input sample and graphed normalized to mock cells with interferon treatment. Statistical significance was calculated using One-Way Anova and represented by asterisks; **p-value <0.01, ***p-value <0.001, ****p-value <0.0001. (C) Fibroblasts were infected (MOI = 1) with a WT virus. At the time of infection, cells were treated with DMSO (vehicle control) or 5 *μ*M Ruxolitinib every 24 hours. At 72 hpi, DNA and RNA was isolated and transcripts encoding UL138 and IE were quantified by RT-qPCR normalized to H6PD. Viral genomes were analyzed through qPCR using primers for b2.7kb and RNaseP as a loading control. Relative expression was determined using the ΔCT method and values were normalized to the DMSO control and graphed for statistical analysis. Statistical significance was calculated using unpaired student t test and represented by asterisks; **p-value <0.01. Graphs represent the mean of three replicates and error bars represent SEM.

Considering the presence of pSTAT1 at RCs at 72 hpi, we hypothesized that pSTAT1 binds to the viral genome to regulate viral gene expression. Utilizing PhysBinder, we identified 86 pSTAT1 consensus sites on the viral genome which include ISRE or GAS. Two sites with an ISRE are within the *UL133-UL138* locus upstream of UL138, designated site 1 and site 2 (Fig 7A). The promoter regions of the ULb’ locus have been minimally characterized [49]. However, several transcripts have been mapped to encode UL138 and the STAT1 binding sites reside upstream of the transcriptional start sites [50,51]. To understand if pSTAT1 was binding to either of these two sites during HCMV infection, we performed CUT&RUN paired with RT-qPCR to quantitatively detect target sequences bound by STAT1 (Fig 7B). CUT&RUN captures DNA bound by a protein using an antibody specific to the protein and micrococcal nuclease conjugated to protein A-protein G to cleave and release the DNA-protein complex [52,53]. We analyzed pSTAT1 binding to the ISRE consensus sequence of the IRF1 ISG in uninfected fibroblasts with or without universal interferon stimulation for 30 minutes, as a positive control for binding. Universal type 1 interferon stimulation increased pSTAT1 binding to the IRF1 consensus sequence relative to unstimulated cells. In WT infection at 48 hpi, we detected pSTAT1 bound at both site 1 and 2 upstream of UL138 relative to the uninfected IRF1 control, whereas we did not detect binding of STAT1 to these sites in Δ*UL138*_STOP_ infection. Therefore, this data suggests pSTAT1 binds to the UL133-UL138 region of the viral genome during HCMV infection in a manner dependent on UL138.

Given pSTAT1 binds to the viral genome at two sites upstream of UL138 during HCMV infection, we next wanted to determine if UL138 gene expression could be regulated by STAT1 signaling. We treated infected fibroblasts in the presence of Ruxolitinib, a JAK1/2 inhibitor and analyzed UL138 expression relative to viral genomes by RT-qPCR when phosphorylation of STAT1 was inhibited (Fig 7C). With inhibition of JAK1/2, UL138 transcripts were diminished by 25% relative to viral genome copy number. By contrast, Ruxolitinib had no effect on immediate early 1 (IE1) expression in a TB40/E infection relative to a vehicle control. This data suggests that JAK1/2 signaling regulates UL138 gene expression.

### USP1 and pSTAT1 are required for the establishment of viral latency

**Fig 8:**
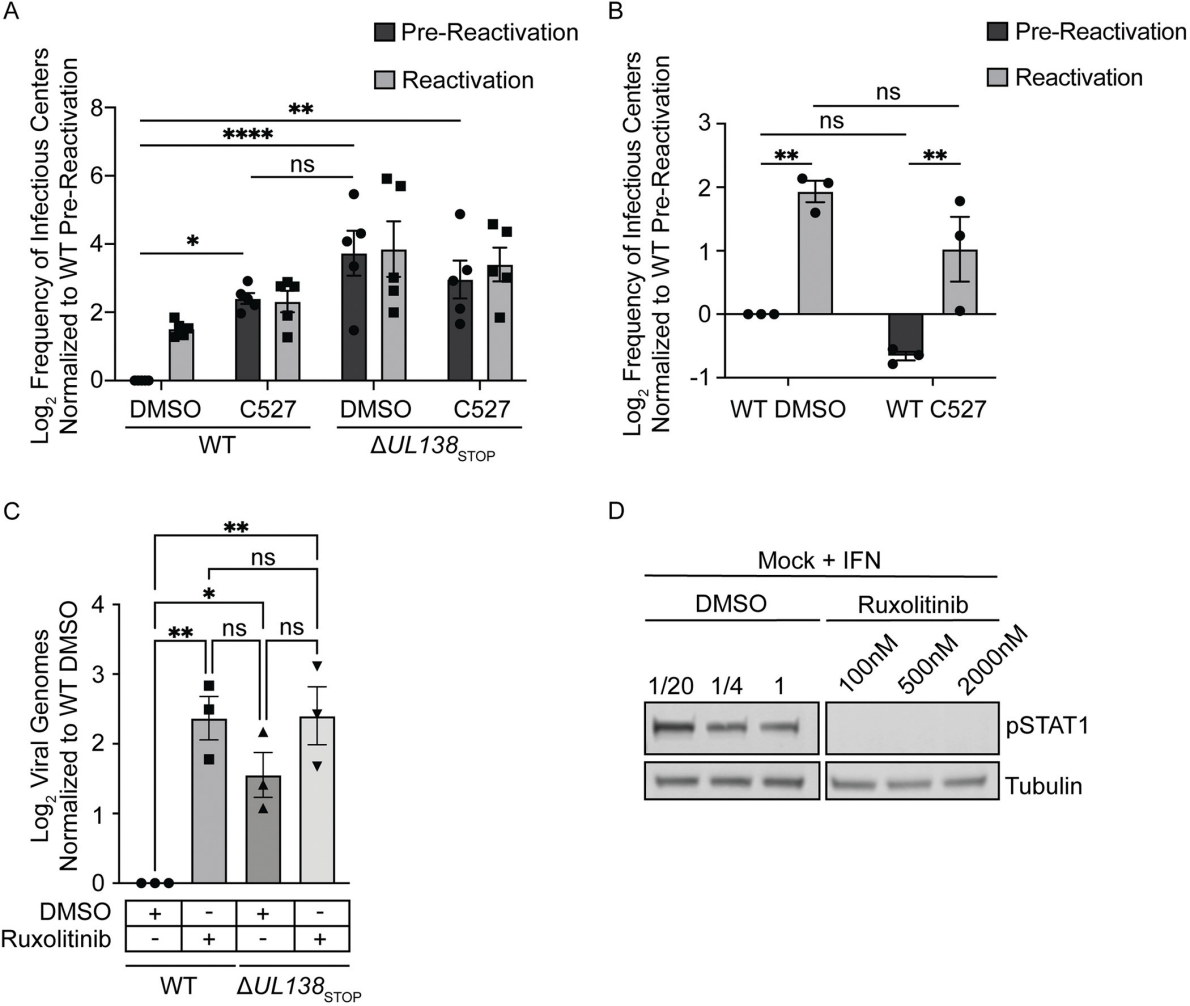
USP1 and pSTAT1 are required for suppression of viral replication and latency establishment. (A) CD34+ HPCs were infected with either WT or Δ*UL138*_STOP_ virus (MOI = 2). At 24 hpi, CD34+/GFP+ cells were sorted and seeded into long-term culture in the presence of DMSO or 1 *μ*M C527. After 10 days in culture, populations were either mechanically lysed (pre-reactivation) or whole cells (reactivation) were plated onto fibroblast monolayers in cytokine-rich media. 14 days later, GFP+ wells were scored and frequency of infectious centers was determined by limited dilution analysis. The frequency of infectious centers was normalized to WT pre-reactivation and the average of 3 independent experiments is shown. Due to donor variability in the Δ*UL138*_STOP_ infection, data was Log2 transformed and graphed. Statistical significance was calculated using Two-Way Anova and represented by asterisks; *p-value <0.05, **p-value <0.01, ****p-value >0.0001. (B) CD34+ HPCs were infected and sorted for long term culture as described in Fig 8A without the addition of C527. At day 10, CD34+ HPCs were plated onto fibroblast monolayers in cytokine-rich media in the presence of DMSO or 1 *μ*M C527. 14 days later, GFP+ wells were scored and frequency of infectious centers was determined by limited dilution analysis. The frequency of infectious centers was normalized to WT pre-reactivation and the average of 3 independent experiments is shown. Statistical significance was calculated using Two-Way Anova and represented by asterisks; **p-value <0.01. Graphs represent the mean of three replicates and error bars represent SEM. (C) CD34+ HPCs were infected with either WT or Δ*UL138*_STOP_ virus (MOI = 2) in cytokine-rich media in the presence of DMSO or 500 nM Ruxolitinib. Every 24 hours, 500 nM Ruxolitinib was refreshed in the media. At days 1 and 5 post infection, DNA was isolated for qPCR using primers for b2.7kb and RNaseP. Viral genomes were determined using ΔCT method and the day 5 values were normalized to the corresponding day 1 values. For graphing, day 5 genomes were normalized to WT DMSO and Log2 transformed. Statistical significance was calculated using Two-Way Anova and represented by asterisks; *p-value <0.05, **p-value <0.01. Graphs represent the mean of three replicates and error bars represent SEM. (D) To ensure Ruxolitinib inhibited pSTAT1, CD34+ HPCs were cultured in the presence of 100 nM, 500 nM, or 2000 nM Ruxolitinib or equivalent DMSO for 3 hours before being treated with 1000 U/mL universal type 1 interferon for 30 minutes and immunoblotted for pSTAT1 and Tubulin.

Given that UL138 has been defined as important to the establishment of latency, we next wanted to investigate the possible role of USP1 in viral latency. We chemically inhibited USP1 activity with C527 in primary CD34+ HPCs infected with WT or Δ*UL138*_STOP_ virus. For these experiments, infected CD34+ HPCs were isolated by fluorescent activated cell sorting and then seeded into long-term bone marrow cultures over a stromal cell support to maintain HPC phenotype and function [54]. At 10 dpi, HPC cultures were split. Half the cells were seeded by limiting dilution onto fibroblast monolayers in a cytokine-rich media to promote myeloid differentiation and HCMV reactivation. The other half of the cells were mechanically lysed and seeded in parallel by limiting dilution to determine virus produced during the culture prior to stimulation of reactivation (pre-reactivation). Fourteen days post seeding, infectious centers (GFP+ wells) were counted to determine the frequency of reactivation using extreme limiting dilution assay (ELDA). The WT virus establishes latency indicated by the low level of infectious centers measured in the pre-reactivation control and the frequency of reactivation increased following stimulation of reactivation. C527 inhibition of USP1 increased virus replication and production of infectious centers in the pre-reactivation control to levels equivalent to that in reactivation in three independent experiments, indicating a failure to establish latency (Fig 8A). Δ*UL138*_STOP_ infection, as shown previously, also fails to establish a latent infection and replicates to levels similar to the WT infection with C527 treatment. Addition of C527 to Δ*UL138*_STOP_ infection had no additional effect on the phenotype, consistent with the UL138-USP1 interaction being a key function of UL138 in the establishment of latency. We next asked if USP1 played any role in reactivation. We infected CD34+ HPCs with a WT virus, as described above; however, we added C527 or DMSO as a vehicle control only at the time of reactivation. Inhibition of USP1 at the time reactivation did not impact infectious centers production (Fig 8B). Therefore, USP1 activity is important for establishing latency, but not for reactivation.

Given the role of USP1 activity in the establishment of latency and the role of the type 1 IFN response in reversible silencing of the genome [55], we next wanted to investigate the role of STAT1 in the establishment of latency. We infected CD34+ HPCs with a WT or Δ*UL138*_STOP_ virus in the presence or absence of the JAK1/2 inhibitor, Ruxolitinib, added at the time of infection. As reported previously [26], the failure of Δ*UL138*_STOP_ to enter latency is reflected in increased viral genome synthesis in CD34+ HPCs relative to the WT infection. Inhibition of JAK1/2 resulted in increased in viral genomes in the WT infection at 5 dpi, reflecting increased viral activity to the level of Δ*UL138*_STOP_ infection, indicating a failure to establish latency (Fig 8C). Ruxolitinib also increased genome replication in Δ*UL138*_STOP_ infection, but more modestly than in the WT infection. To ensure Ruxolitinib was inhibiting the phosphorylation of STAT1, uninfected CD34+ HPCs were treated with 1000 U/mL universal type 1 interferon in the presence of differing doses of DMSO or Ruxolitinib (Fig 8D). JAK1/2 inhibition diminished pSTAT1 at all doses, however, 500 nM was chosen because it was the highest dose that didn’t alter proliferation and survival of CD34+ HPCs. Taken together, these results are consistent with UL138-USP1 modulation of STAT1 to repress viral replication for latency. Further work is required to define STAT1 binding to the viral genome and its regulation of viral gene expression.

## Discussion

Type 1 interferons, IFN*α* and IFN*β*, bind to IFNAR and stimulates JAK1/2-mediated phosphorylation of STAT1 and STAT2 along with recruitment of IRF9 to generate the ISGF3 complex [56]. The ISGF3 complex translocates to the nucleus and binds to a consensus ISRE to promote the of expression of many ISGs to limit viral infection [57]. However, herpesviruses encode multiple mechanisms to manipulate the innate immune response to prevent restriction. HCMV combats elimination by the innate immune response by inhibiting the signaling cascade at multiple checkpoints during a replicative infection [58–60]. Infection of fibroblasts and epithelial cells with the high-passage HCMV strain, Towne, causes proteasomal degradation of JAK1 to terminate phosphorylation of STAT1 [43,59]. In addition, IE1 has been found to interact with STAT1 and STAT2 and prevent DNA binding of the ISGF3 complex to prevent ISG54 and Mx1 expression [61]. However, these studies were performed with a laboratory-adapted strain of HCMV that lacks genes in the UL*b’* locus, including UL138. In addition to thwarting IFN signaling to ensure virus replication, HCMV may also commandeer it. HCMV has been reported to stimulate pSTAT1 [62] and hijack the innate immune DNA sensor, IFI16, to activate the viral major immediate early promoter (MIEP) [63]. In addition, exogenous IFN*β* addition has been shown to reversibly silence the MCMV genome [55]. Further, HCMV latently-infected CD14+ monocytes exhibit a pro-inflammatory profile of gene expression, but are blunted in their response to type I or II triggers of STAT1 signaling [64]. Therefore, despite the anti-viral, nature of a type-I IFN response, HCMV may commandeer this response for persistence [65].

Here we have demonstrated a mechanism by which UL138-host interactions stimulate pSTAT1 for the establishment of latency. Notably, we have found that UL138 interacts with UAF1 and USP1 (Table 1 and Fig 1). The scaffold protein UAF1 forms a complex with USP1, USP12, and USP46 that is essential for their activity. UAF1-USP1 in best understood for its role in regulating nucleation of DNA damage responses [29,30,33]. The UAF1 (WDR48) interaction with UL138 has been previously reported [35,36]. While Nobre et al. [36] did not follow up this interaction, Li et al. [35] reported that UAF1 (WDR48) was important for viral genome synthesis and virus replication in MRC5 cells infected with the Han strain. We find that inhibition of USP1 with C527 increased TB40/E genome replication in CD34+ HPCs, suggesting that at least the UAF1-USP1 complex is suppressive to viral genome replication, consistent with the role of UL138 in suppressing replication [17]. We did not directly test the role of the UAF1 scaffold in HCMV infection, as its knockdown is likely to have pleiotropic effects due to the combined loss of USP1, USP12, and USP46 activity. In HPV infection, viral helicase, E1, promotes UAF1-USP1 activity for viral genome replication [66,67] and the high-risk HPV oncogenes, E6 and E7, delay UAF1-USP1 mediated deubiquitination of the Fanconi anemia (FA) DNA crosslink repair, contributing to genomic instability [68]. Further studies are required to address the mechanisms by which UL138-UAF1-USP1 regulate HCMV replication and genome synthesis.

USP12 also co-precipitated with UL138 (Table 1), suggesting that in addition to USP1, that UL138 may also regulate USP12. USP12 has been implicated in sustaining pSTAT1 by binding to CBP and preventing acetylation of pSTAT1 and subsequent dephosphorylation by PTPN2 [69]. Although the role of the UL138-UAF1-USP12 complex will be addressed in future studies, the inclusion of USP12 in the IP/MS data set suggests an additional mechanism by which UL138 sustains pSTAT1. In support, JAK1 was also found a low-ranking hit identified in the IP/MS and bolsters the conclusions that UL138 interacts with host proteins to regulate STAT1 signaling; however we found no evidence of UL138 modulating JAK1 levels or activation (Fig 4E).

UAF1-USP1 has been reported to sustain pSTAT1 signaling by deubiquitinating and preventing proteasomal degradation of TBK1 [34]. While we show that pSTAT1 depends on UL138 and USP1 at late times during infection in fibroblasts (Figs 2 and 3), we were surprised that neither total or phosphorylated levels of TBK1 were affected by UL138 or USP1 as a possible mechanism to sustain pSTAT1 (Fig 4). This suggests that while both our studies and those of Yu et al. [34] demonstrate a role for USP1 sustaining pSTAT1, it may not be through the rescue of TBK1 from turnover (Fig 4A). UL138 does not affect the activation or levels of kinases upstream of STAT1 phosphorylation (Fig 4E) or two phosphatases of pSTAT1, PTPN2 and SHP-2 [44,45] (Fig 4F). Another study found that pSTAT1 could be targeted for proteasomal degradation in the nucleus to terminate pSTAT1 signaling [46]. However, although we show that pSTAT1 is turned over via the proteasome during HCMV infection, UL138 does not have an effect on proteasomal turnover (Fig 4F).

It has been reported that STAT1 must be deubiquitinated at Lys511 and Lys652 to license its recruitment to IFNAR and subsequence phosphorylation by JAK1/2 [70]. Therefore, it is possible that the UAF1-USP1 complex might deubiquitinate STAT1 to promote its recruitment to and subsequent activation by IFNAR. It will be important to determine if this is the mechanism by which USP1 and UL138 function to sustain STAT1 signaling. As USP12 has been reported to antagonize PTPN2’s dephosphorylation of pSTAT1, it is also possible that interaction with USP12 contributes to UL138-mediated sustainment of pSTAT1 during HCMV infection. Sustainment of pSTAT1 by USP12 may be coordinated with that of USP1 in the context of HCMV infection or they may function distinctly under different contexts. Future studies will investigate these possibilities to define the mechanisms by which UL138-complexes sustain STAT1 activity during HCMV infection.

Contrary to our findings, Kalejta and colleagues showed that UL138 inhibits the innate immune response by targeting STING for degradation [42]. However, UL138-mediated loss of STING was only evident when both UL138 and STING were transiently overexpressed. Consistent with our findings (Fig 4C), no UL138-dependent changes in STING protein levels were observed during infection. However, Kalejta and colleagues went on to show increased IFNβ and CXCL10 transcripts in *UL138*-M16_STOP_ infection in CD34+ HPCs, concluding that UL138 functions in immune evasion, degrading STING to abate a type-1 interferon response. However, an alternative explanation of the increase in IFNβ and CXCL10 expression is derived from the fact that transcripts were analyzed from unsorted hematopoietic cells. As UL138-mutant viruses are more replicative, increased levels of ISGs in a mixed population of infected and uninfected cells may be due to the enhanced replication of the mutant virus or the response of uninfected cells in the culture to secreted type 1 IFNs. Our results are consistent with that of the Kalejta laboratory in that changes in innate signaling in infection appear to occur independently of changes in total or phosphorylated levels of TBK1 and STING.

We show that the prolonged pSTAT1 stimulated during HCMV infection correlates with an enhanced and sustained expression of several type 1 IFN-associated ISGs in a UL138-dependent manner. These include MX1, OAS, IFIT1, and IFIT2 (Fig 5B). Type 2 interferons also lead to the phosphorylation of STAT1, which form homodimers which bind to GAS to drive expression of ISGs that are similar and distinct to type 1 IFN driven ISGs [47]. However, we found no induction of a type-2 IFN driven ISG, ICAM1, above uninfected levels. In both WT and Δ*UL138*_STOP_, the induced type 1 IFN ISGs diminish by 72 hpi despite sustained pSTAT1. This is undoubtedly due, in part, to robust inhibition of type 1 IFN signaling by multiple HCMV gene products [60,71–74]. However, we also show that pSTAT1 localizes to nuclear viral RCs (Figs 6 and S2) and binds to the viral genome to stimulate UL138 expression (Fig 7). With respect to regulation of viral gene expression, STAT1 signaling been shown suppress lytic reactivation of MHV-68. The MHV-68 ORF50 promoter, which is essential for the lytic switch, encodes STAT1 repressive elements to negatively regulate lytic replication in response to IFNγ [75]. By contrast, our findings suggest that HCMV commandeers STAT1 to stimulate the expression of a pro-latency gene—achieving the same end as MHV-68, but through a distinct mechanism. Further, it is possible that sequestration of pSTAT1 in viral RCs during HCMV infection, may also result in a distinct or diminished expression profile of ISGs at late times in infection. Our data also suggests a lack of paracrine signaling (Fig 6) as the induction of pSTAT1 is limited to HCMV infected cells and not observed in neighboring uninfected cells. This coincides with the observation that activation of STAT1 is limited to MCMV infected cells [76] and that antiviral responses (e.g. IFN, ISGs) are dampened in neighboring cells [77]. Trilling et al; shows STAT1 and STAT3 activation fails to induce IRF1, SOCS1, and SOCS3 expression and they concluded MCMV infection diminishes STAT1/STAT3 signaling downstream of activation. Similarly, our work has shown a diminishment of ISG induction at late times post infection which correlates with localization of pSTAT1 to viral replication compartments. In addition, we observe USP1 and JAK1/2 dependent effects on viral replication and gene expression, suggesting an important role for these pathways in regulating viral replication and latency.

UL138 has been shown to contribute to latency by stimulating the expression of the host transcription factor, EGR-1 [18], through MEK/ERK signaling. EGR-1 binds the viral genome upstream of UL138 to stimulate its expression for latency. Furthermore, inhibition of JAK1/2 activity in HCMV infected CD14+ monocytes increases viral transcripts [3]. Similarly, our findings here show that UL138 interaction with USP1 also regulates UL138 gene expression through sustaining activity of STAT1. Inhibition of EGFR or STAT1 with chemical inhibitors results in a loss of latency and increases virus replication in the absence of a stimulus for reactivation. Future studies will investigate more globally the changes in viral gene expression driven by EGR-1 and pSTAT1 to influence the latency program in CD34+ HPCs.

Viruses excel at manipulation of host signaling and innate pathways to evade elimination. However, as illustrated by our study, viruses also commandeer host defenses to their benefit, for example, in the case of viral persistence. Inhibition or loss of type 1 IFN signaling prevents the establishment of HIV-1 latency in macrophages [78] and murine gammaherpesvirus 68 (MHV-68) latency in splenocytes [79]. Epstein-Barr virus (EBV) latent membrane protein 1 (LMP-1) stimulates STAT1 activity mediated through NF*κ*B signaling [80]. Further, EBV EBNA1, which is important for the maintenance of the EBV episome in latency, was shown to enhance the expression of STAT1 [81]. This induction sensitized cells to IFNγ treatment, although basal levels of ISGs were not changed, possibly due to LMP-2-suppression of STAT1 signaling. STAT1 has also been shown to positively regulate the BamHI-Q promoter since interference with Jak/STAT signaling reduces BamH1-Q promoter activity [82]. In the context of EBV transformed B cells, STAT1 was shown to be important to the latency III viral program and to differentially regulate viral gene expression in tumors [83,84]. Downregulation of STAT1 in this context resulted in increased viral lytic gene expression and lytic cycle entry, which is accompanied by downregulation of major histocompatibility complexes (MHC) class I and II at the cell surface. Interferon regulatory factors (IRF) also play complex roles in regulating viral gene expression. Interferon regulatory factor 2 (IRF2) and 7 (IRF7) bind and repress the BamHI-Q promoter of EBV to promote type III latency establishment [85,86]. IRF7 is induced in a TRAF6- and RIP1-dependent manner by LMP-1 of EBV [87–90]. By contrast, IRF8, which is primarily expressed in hematopoietic cells, has been shown drive the expression of EBV lytic genes, such as BGLF2, and that the PIAS1 negative regulator of STAT1 signaling is important for latency [91]. In alphaherpesviruses, the viral E3 ubiquitin ligase, ICP0, of herpes simplex type 1 (HSV-1) antagonizes STAT1 for replication. In the absence of ICP0, STAT1 restricts virus replication, suggesting a possible role for STAT1 in the establishment of HSV-1 latency [92]. STAT1 could regulate expression of the HSV-1 latency associated transcript (LAT), as binding sites have been mapped [93] and IFNβ modulates LAT expression and neuron survival [94]. While herpesviruses appear to largely commandeer innate signaling for the establishment of latency or to restrict lytic reactivation, it is also clear that this must be carefully controlled and some latency-associated viral factors may inhibit innate responses, such as MHV68 M2 downregulating both STAT1 and STAT2 [95].

There are many points of crosstalk between the DNA damage response and innate immune response [96]. The inflammatory response induced by the innate immune response has consequences on both pathogens and host cells. A byproduct of the innate immune response includes generation of reactive oxygen species (ROS) and nitrogen species (RNS) which can lead to host DNA damage [97]. Additionally, it’s been proposed that prolonged elevated inflammation can lead to an accumulation of unrepaired DNA damage [98]. The reverse is also true where the DNA damage response can lead to cytosolic DNA, including mitochondrial DNA, that can trigger an innate immune response through the classic innate immune DNA sensors [99,100]. Future work will investigate UL138’s regulation of UAF1/USP1 as a potential mechanism to mitigate cell stress during a sustained innate immune response or if the proteins involved in the DNA damage response have an independent role in HCMV infection. While UL138 may modulate STAT1 activity to regulate innate responses for latency, it is also possible that STAT1 plays other critical roles central to latency and reactivation, such as its lesser understood role in hematopoietic differentiation that may occur independently of ISGs [101–105] or as a transcription factor recruited for viral gene expression. It will also be important to differentiate these possibilities in future work and to understand the role of other UAF1-USP complexes in the establishment of latency.

## Materials and methods

### Cells

MRC-5 lung fibroblasts (ATCC), HEK293T/17 cells (ATCC), Sl/Sl stromal cells (Stem Cell Technology), M2-10B4 stromal cells (Stem Cell Technology), and CD34^+^ HPCs were maintained as previously described [19,26]. Human CD34^+^ HPCs were isolated from de-identified medical waste following bone marrow isolations from healthy donors for clinical procedures at the Banner-University Medical Center at the University of Arizona. Latency assays were performed as previously described [19,26]. In chemical treatments of MRC-5 lung fibroblasts, 0.88 *μ*M C527 (ApexBio), 5 *μ*M Ruxolitinib (STEMCELL Technologies) every 24 hours, or 1000 U/mL universal type 1 interferon (R&D Systems) for 30 minutes. CD34+ HPCs were treated with 1 *μ*M C527 or every 24 hours with 500 nM Ruxolitinib.

### Viruses

Bacterial artificial chromosome (BAC) stocks of TB40/E WT virus, a gift from Christian Sinzger [106], was engineered to express GFP from an SV40-promoter [17]. Creation of Δ*UL138*_STOP_ virus is as previously characterized [37]. Fibroblasts were infected at an MOI of 1 unless otherwise specified in the figure legend. CD34+ HPCs were infected at an MOI of 2.

### Mass spectrometry

To identify pUL138 interacting partners, fibroblasts were infected at an MOI of 3 with TB40/E-*UL138*_*3XFLAG*_. Interacting proteins were isolated by cryogenic cell lysis at 48 hpi and rapid immunoaffinity purification as described previously [19,107]. Briefly, protein complexes were isolated by immunoprecipitation for 1 hr. using anti-FLAG antibody conjugated to Dynabeads (ThermoFisher Scientific). Proteins were eluted, dried and resuspended in SDS-loading buffer. Samples were alkylated with iodoacetamide and separated by SDS 10% PAGE. The entire lane was cut into sections and subjected to in-gel tryptic digestion. The isolated tryptic peptides were analyzed by LC-MS/MS using an ESI-LTQ XL mass spectrometer (ThermoScientific). Peptide identification was carried out using SEQUEST with a global false discovery rate of 5%.

### Plasmids and siRNAs

USP1_HA_ was purchased from Addgene (BC050525). UL138_myc_ was overexpressed in pLVX_TetONE_puro vector and UAF1_HA_ was expressed from a pCIG vector. siGenome SMARTpool siRNAs targeting USP1 and nontargeting control (NTC) were purchased from Dharmacon. siRNAs were reverse transfected [108] into fibroblasts using Lipofectamine RNAiMAX reagent (Thermo Fisher Scientific) according to the manufacturer’s instructions. The next day, media was exchanged. 48 hours later, fibroblasts were infected (MOI = 1) for 24, 48, and 72 hpi.

### Immunoblotting

Lysates were separated by electrophoresis on precast 4–20% precast gels (BioRad). Gels were transferred onto Immobilon-P PVDF membrane (EMD Millipore). Antibodies were incubated in with a blocking solution of 5% BSA in TBS-T, as per antibody manufacturer specifications. After antibody staining, blots were incubated with fluorescent secondary antibodies and imaged and quantitated using a Li-Cor Odyssey CLx infrared scanner with Image Studio software. Antibodies and sources are defined in Table 2.

**Table 2:** Antibody description and sources.

Antibody	Species	Source	Concentration
pSTAT1 (Y701)	Rabbit	Cell Signaling	WB 1:1000 IF 1:50
STAT1	Rabbit	Cell Signaling	WB 1:1000
STAT1	Mouse	Cell Signaling	WB 1:1000
pSTING (S366)	Rabbit	Cell Signaling	WB 1:1000
STING	Rabbit	Cell Signaling	WB 1:1000
pTBK1 (S172)	Rabbit	Cell Signaling	WB 1:1000
TBK1	Rabbit	Cell Signaling	WB 1:1000
JAK1	Rabbit	Cell Signaling	WB 1:1000
pJAK1 (Y1022/1023)	Rabbit	Cell Signaling	WB 1:1000
TYK2	Rabbit	Cell Signaling	WB 1:1000
pTYK2 (Y1054/1055)	Rabbit	Cell Signaling	WB 1:1000
SHP-2 (PTPN11)	Rabbit	Cell Signaling	WB 1:1000
pSHP-2 (pPTPN11) (Y542)	Rabbit	Cell Signaling	WB 1:1000
PTPN2	Rabbit	Cell Signaling	WB 1:1000
Myc epitope tag	Rabbit	Cell Signaling	WB 1:1000
HA epitope tag	Rabbit	Cell Signaling	WB 1:1000
USP1	Rabbit	Cell Signaling	WB 1:1000
IE1/2 (3H4)	Mouse	Tom Shenk; Princeton University	1:200
Tubulin (DM1A)	Mouse	Sigma Aldrich	1:2000
UL44	Mouse	Virusys	IF 1:12000
DAPI dilactate	N/A	Sigma Aldrich	1:2000
Alexa Fluor 546 anti rabbit	Goat	Molecular Probes	1:5000
Alexa Fluor 647 anti mouse	Goat	Molecular Probes	1:5000

### Immunofluorescence

Cells were grown on coverslips and infected at an MOI of 1 with TB40/E or Δ*UL138*_STOP_ virus. Uninfected cells were treated with DPBS or 1000 U/mL of universal type 1 interferon (R&D Systems) for 30 minutes. At each time point, cells were prepared for immunofluorescence by the cell signaling protocol for the pSTAT1 antibody. pSTAT1 and UL44 were detected through monoclonal antibodies and secondary antibodies were conjugated to Alexa Fluor 546 (green) or 647 (red). Coverslips were imaged using a DeltaVision deconvolution microscope.

### Subcellular fractionation

Fractionation was performed as described previously [109]. Fibroblasts were infected (MOI = 1) and trypsinized and washed in PBS at each time point. Cells in suspension were then sequentially lysed in buffers containing detergents to separate subcellular compartments: 25 μg/mL digitonin (cytosolic fraction), 1% NP-40 (membrane-bound fraction), and 0.5% sodium deoxycholate and 0.1% SDS with 25 U/mL Benzonase Nuclease (nuclear fraction).

### RT-qPCR and qPCR

Cells were infected with 1 MOI of TB40/E_GFP_ and DNA and RNA was isolated using Quick-DNA/RNA miniprep kit (Zymo Research) from 0–72 hpi. RNA was reverse transcribed into cDNA using SuperScript VILO cDNA Synthesis kit (Thermo Fisher Scientific). cDNA and DNA was quantified using LightCycler SYBR Mix kit (Roche) and corresponding primers (Table 3). Assays performed on Light Cycler 480 and corresponding software. Relative expression was determined using the ΔΔCT method normalized to H6PD. Viral genomes were quantified using a standard curve and normalized to RNAseP. For quantification of ISGs, fibroblasts were infected (MOI = 1) for 24, 48, and 72 hpi. RNA was isolated using Quick-DNA/RNA miniprep kit (Zymo Research) and cDNA was prepared using 1000 ng of total RNA and random hexamer primers (Thermo Fischer Scientific). Real-time qPCR (TaqMan) was used to analyzed cDNA levels with specific TaqMan primer and probe sets (Thermo Fisher Scientifc). Relative expression was determined using the ΔΔCT method using GAPDH as the standard control.

**Table 3:** Primer sequences.

Primer	Sequence
UL138 RT-qPCR Forward	5’-TGAGATCTTGGTCCGTTGG-3’
UL138 RT-qPCR Reverse	5’-GTGTGTTATCCGCGACGAC-3’
IE1 RT-qPCR Forward	5’-TGACCGAGGATTGCAACGA-3’
IE1 RT-qPCR Reverse	5’-CCTTGATTCTATGCCGCACC-3’
H6PD RT-qPCR Forward	5’-GGACCATTACTTAGGCAAGCA-3’
H6PD RT-qPCR Reverse	5’-CACGGTCTCTTTCATGATGATCT-3’
RNAseP Forward	5’-GACGGACTGCGCAGGTTA-3’
RNAseP Reverse	5’-CCATGCTGAAGTCCCATGA-3’
IRF1 CUT&RUN Forward	5’-CCCTTCGCCGCTAGCTCTA-3’
IRF1 CUT&RUN Reverse	5’-GCTGCGTGCCGTCATTTC-3’
Site 1 CUT&RUN Forward	5’-GACATTCCCAGAACCGGTG-3’
Site 1 CUT&RUN Reverse	5’-CAAGGGCGTGGAGATGCCAG-3’
Site 2 CUT&RUN Forward	5’-GAATTCCCGGTAACGGATGAG-3’
Site 2 CUT&RUN Reverse	5’-CGAATACGTTTTCGGGACCC-3’

### CUT&RUN

For fibroblasts, 1e5 cells were utilized per condition. Cells were infected (MOI = 1) with WT or Δ*UL138*_STOP_ virus for 48 hours and then processed for CUT&RUN (Cell Signaling Technologies) as per manufacturer’s recommended protocol. pSTAT1, Histone H3 (positive control), and normal mouse IgG (negative control) were pulled down using antibodies. Isolation of DNA was performed through phenol chloroform extraction as per the manufacturer’s protocol. qPCR was performed with LightCycler SYBR Mix kit (Roche) and primers to pSTAT1 binding sites 1, 2, and IRF1 (Table 3). Relative expression was calculated against a 2% input control. Samples were then normalized to uninfected cells stimulated with 1000 U/mL universal type 1 interferon for 30 minutes (R&D Systems).

### Viral latency and reactivation

Infectious centers were quantitated in CD34^+^ HPCs, as described previously (39). Frequency of infection centers were calculated using extreme limiting dilution analysis (42). For the investigation of USP1 activity during latency establishment, CD34^+^ HPCs were treated with 1 *μ*M C527 (ApexBio) after sorting for CD34^+^ GFP^+^ populations and when stromals were replaced at 6 dpi. The study for USP1’s role in reactivation, 1 *μ*M C527 was added at the time of reactivation. Proliferation of CD34^+^ cells during chemical inhibition was calculated by observing the fold change in the number of cells prior to and after inhibition for each condition. To understand JAK1/2’s role in latency establishment, CD34+ cells were treated with 500 nM Ruxolitinib (STEMCELL Technologies) every 24 hours for 5 days. Cells were lysed and DNA was extracted at days 1 and 5 utilizing the Quick-DNA/RNA Miniprep (Zymo Research). Viral genomes were quantified using a standard curve and normalized to RNAseP.

### Statistical analysis

All statistics were calculated using GraphPad Prism version 7 software. Statistics for experiments in this study were calculated using either unpaired student T-test or analysis of variance (ANOVA) for statistical comparison, which is indicated in the figure legends with asterisks representing statistical significance; *p-value <0.05, **p-value <0.01, ***p-value <0.001, ****p-value <0.0001.

## Supporting information

S1 Data Excel spreadsheet containing the numerical data used for generating graphs in separate sheets. (XLSX)

S1 Table pUL138 interacts with proteins involved in the regulation of STAT1 signaling and the DNA damage response. Candidate interactions determined by IP/MS from infected fibroblasts (TB40/E-*UL138*_FLAG_) (MOI = 3) at 48 hpi. UL138_FLAG_ was immunoprecipitated with a Flag antibody and following tryptic digest, peptides were identified by IP-MS/MS. Interacting candidates were subtracted from a control Flag pull-down from infected fibroblasts without a Flag tagged UL138. 128 candidates were identified and top interactions were identified by STRING and NCBI analysis. Top-ranking candidates involved in STAT1 signaling are listed based on the peptide count, scan count, and percent coverage.
(XLSX)

S1 Fig USP1 knockdown does not alter TBK1 levels during HCMV infection. Fibroblasts were reverse transfected with 3 combined siRNA for a non-targeting control (NTC) or USP1. 24 hours post reverse transfection, the media was replenished. 48 hours post reverse transfection, fibroblasts were infected (MOI = 1) with either a WT or Δ*UL138*_STOP_ virus and lysates were immunoblotted to detect USP1, TBK1, and Tubulin with antibodies.
(TIF)

S2 Fig pSTAT1 localizes to sites of viral DNA synthesis and transcription as early as 48 hpi. Fibroblasts plated on glass coverslips and infected (MOI = 1) for 48 hpi with a WT or Δ*UL138*_STOP_ virus. Cells were prepared for immunofluorescence per the antibody manufacturer’s instructions and imaged with a DeltaVision deconvolution microscope.
(TIF)
