# Supplementary figures and images for: Human cytomegalovirus UL138 interaction with USP1 activates STAT1 in infection
Source: PLoS Pathog. 2023 Jun 8;19(6):e1011185. doi: 10.1371/journal.ppat.1011185 (PMC10284425; doi:10.1371/journal.ppat.1011185)

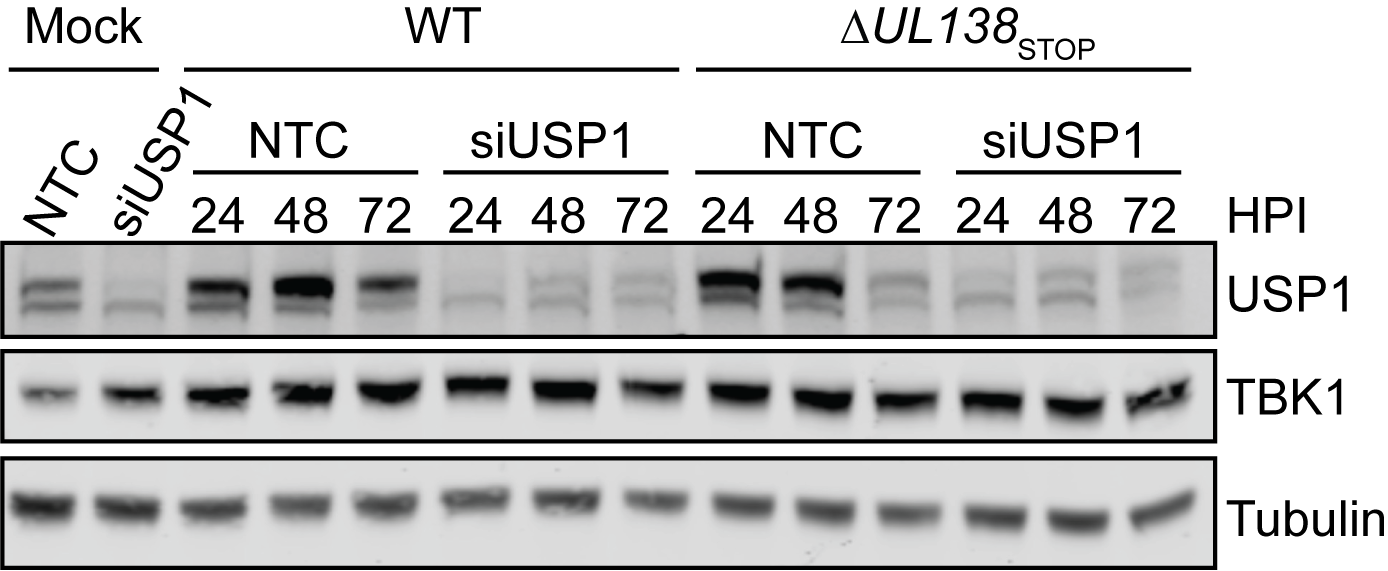

Supplement: S1 Fig — Fibroblasts were reverse transfected with 3 combined siRNA for a non-targeting control (NTC) or USP1. 24 hours post reverse transfection, the media was replenished. 48 hours post reverse transfection, fibroblasts were infected (MOI = 1) with either a WT or ΔUL138STOP virus and lysates were immunoblotted to detect USP1, TBK1, and Tubulin with antibodies. (TIF) [file ppat.1011185.s003.tif]

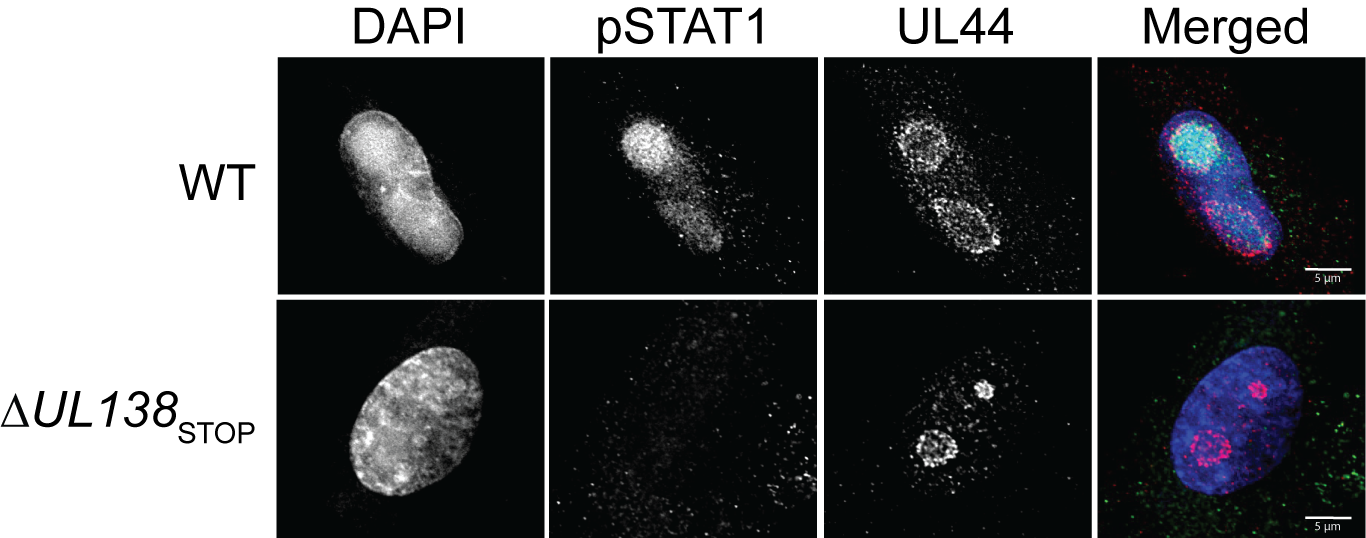

Supplement: S2 Fig — Fibroblasts plated on glass coverslips and infected (MOI = 1) for 48 hpi with a WT or ΔUL138STOP virus. Cells were prepared for immunofluorescence per the antibody manufacturer’s instructions and imaged with a DeltaVision deconvolution microscope. (TIF) [file ppat.1011185.s004.tif]
